# Hospital-based ciprofloxacin use evaluation in Eastern Ethiopia: a retrospective assessment of clinical practice

**DOI:** 10.11604/pamj.2021.38.62.21626

**Published:** 2021-01-19

**Authors:** Tigist Gashaw Tekalign, Mekonnen Sisay Shiferaw, Tewodros Tesfa Hailegiyorgis, Yohannes Baye Embiale, Firehiwot Amare Abebe

**Affiliations:** 1 Department of Pharmacology and Toxicology, School of Pharmacy, College of Health and Medical Sciences, Haramaya University, P.O. Box 235, Harar, Ethiopia,; 2 Department of Microbiology, Medical Laboratory Sciences, College of Health and Medical Sciences, Haramaya University, P.O. Box 235, Harar, Ethiopia,; 3 Department of Pediatrics and Neonatal Nursing, School of Nursing and Midwifery, College of Health and Medical Sciences, Haramaya University, P.O. Box 235, Harar, Ethiopia,; 4 Department of Clinical Pharmacy, School of Pharmacy, College of Health and Medical Sciences, Haramaya University, P.O. Box 235, Harar, Ethiopia

**Keywords:** Ciprofloxacin, evaluation, appropriateness, indication, duration

## Abstract

**Introduction:**

ciprofloxacin is a second-generation fluoroquinolone, which has been used as one of the top three antibacterial agents prescribed in Ethiopia. However, its use has deviated from the recommendation of standard treatment guidelines resulting in a gradual increase in antimicrobial resistance. Therefore, this study aimed to evaluate the annual use of ciprofloxacin in 2016 based on the standard Ethiopian treatment and World Health Organization guidelines, in governmental hospitals, in Eastern Ethiopia from 1^st^ May to 30^th^ June 2018.

**Methods:**

a hospital-based retrospective cross-sectional study was conducted to evaluate medical records of patients who had taken ciprofloxacin in 2016. The total sample size (n=522) was proportionally allocated to each hospital based on the respective consumption data. A simple random sampling method was employed to collect the required sample. The collected data were entered into SPSS version 21 and analyzed using descriptive analysis.

**Results:**

in this study, 522 medical records were reviewed, with a male to female ratio of 1.03: 1. Ciprofloxacin was indicated in 478 (91.6%) participants whose age was greater than eighteen years. The majority were treated in the medical and emergency outpatient departments (n=477, 91.4%). Urinary tract infections (n=224, 42.9%), acute febrile illnesses (n=68, 13.0%), and typhoid fever (n=54, 10.4%) were the top indications to which ciprofloxacin was prescribed. Non-steroidal anti-inflammatory drugs (NSAIDs) (n=241, 34.7%) and antimicrobials (n=135, 19.6%) were among the most frequently co-indicated agents. Based on the standard Ethiopian treatment guidelines, therapy was appropriate in 30% (n= 159) of patients. The major reason for inappropriate utilization (95%) was the wrong duration of antibiotic use (n=228). Evaluation based on World Health Organization criteria showed that indication, dose, and frequency were in line with the recommendation.

**Conclusion:**

ciprofloxacin was primarily indicated for urinary tract infections. The drug was appropriately used in less than one-third of patients, with the wrong duration being the main reason for overall inappropriate utilization. This trend may potentially impose a high risk to the emergence of drug-resistant microorganisms. To this end, further studies addressing the susceptibility pattern of bacterial isolates towards ciprofloxacin should be carried out.

## Introduction

Since the 1980s, the discovery of fluoroquinolones has become an essential therapeutic breakthrough in chemotherapy. Fluoroquinolones possess broad-spectrum bactericidal properties with a wide safety margin and relatively low risk of resistance. Among them, ciprofloxacin is a second-generation 6-fluoroquinolone with favorable pharmacokinetic properties and a better safety profile [1,2]. Fluoroquinolones, including ciprofloxacin, target bacterial topoisomerase enzymes such as topoisomerase II (DNA gyrase) in gram-negative and topoisomerase IV primarily in gram-positive bacteria. These quinolones inhibit topoisomerase mediated DNA supercoiling at therapeutic concentrations and entail a concentration-dependent bactericidal effect. Mutations of these target enzymes can confer resistance to quinolones [3]. In Ethiopia, ciprofloxacin is used for various diseases, including bacillary dysentery, gastroenteritis, pneumonia, typhoid fever, chancroid, and gonorrhea [4]. It is also one of the commonly used antibiotics in other parts of the world [5-7]. The dominant factor in the emergence and spread of antibiotic-resistant bacterial pathogens is the extensive use of antibiotics [8,9]. Careful and well-focused use of antimicrobials, together with procedures to measure utilization patterns, may provide a chance of preserving the efficacy of antibiotics [10]. In Ethiopia, reports indicate wide practices of inappropriate antimicrobials use by different stakeholders [11-13]. One mechanism to monitor antimicrobial utilization patterns is to carry out a drug use evaluation (DUE) study, a systematic approach that enables assessment of the appropriateness, safety, and effectiveness of a medication to improve patient care [14-20]. Therefore, the objective of this study was to evaluate ciprofloxacin use in 2016 based on standard Ethiopian treatment guidelines and WHO set criteria at selected governmental hospitals: Hiwot Fana Specialized University Hospital (HFSUH), Jugel Hospital (JH), Federal Harar Police Hospital (FHPH) and South-East command III Hospital (SECIIIH) in Eastern Ethiopia.

## Methods

**Study setting, design and population:** Harar is located 526km east of Addis Ababa, the capital of Ethiopia to the East. Harari region is one of the nine national, regional states of Ethiopia, with the town of Harar as its capital [21]. The study was conducted in four governmental hospitals, namely HFSUH, JH, FHPH, and SECIIIH. A cross-sectional retrospective study design was employed to evaluate ciprofloxacin utilization patterns using medical records of patients who had taken the medication drug in by the year 2016. The data were collected from 1^st^ May to 30^th^ June 2018.

**Sample size determination and sampling techniques:** the sample size was determined by using the single population proportion formula:

$$\text{n=}\frac{z_{\alpha }^{2}p(1-p)}{W^{2}}$$

Where: n = sample size, Z = the standard normal value at 95% confidence level, P = point estimate of population, W = maximum width tolerable error, q = 1-p (failure rate). To determine the sample size, the Z statistics value = 95% (1.96) with two-sided tolerable error (α) (W = 5% or 0.05) and P = 50% (0.5) were substituted in the above formula. P-value was taken as 50% for determining an adequate sample size since there was no attempt done to conduct research on this topic and no vivid information about this in the study area.

$$\text{n=}\frac{{\left(1.96\right)}^{2}*0.5(0.5)}{{\left(0.05\right)}^{2}}$$

n = 384. To obtain a greater point estimate contingency was considered to come up with a final sample size of 522.

**Sampling techniques:** the aforementioned sample size was proportionally allotted by multiplying the ratio of each hospital ciprofloxacin consumption data to the total consumption. Accordingly, 209, 183, 63, and 67 ciprofloxacin containing medical records were distributed to HFSUH, JH, SECIIIH, and FHPH, respectively. Simple random sampling was employed to collect the sampling units from the sampling frame, which was prepared from the card numbers of prescriptions containing ciprofloxacin.

**Study variables:** ciprofloxacin use evaluation; sociodemographic status of the patient; indication, dose, frequency and duration of ciprofloxacin; co-administered medications; duration of inpatient hospital stay.

**Inclusion and exclusion criteria:** medical records of patients with prescriptions for ciprofloxacin were included, and records with missing information in more than one criterion stated for analyzing appropriateness as well as with incomplete sociodemographic status were excluded.

**Data collection:** a preliminary study was undertaken on 5% of medical records at Haramaya Hospital, and minor adjustments were made to the data abstraction format. Then, data were collected from patient medication records retrospectively using a structured data abstraction format.

**Data processing and analysis:** the collected data were entered into the statistical package for the social sciences, version 21.0 software (SPSS Inc., Chicago, IL, USA) and checked for clarity. Descriptive methods of analyses (frequency and percentages) were used to summarize the socio-demographic, clinical, and drug-related factors. Besides, for ciprofloxacin utilization, the standard treatment guideline (STG) of Ethiopia was employed by taking the four primary major criteria: i.e. indication for use, dose, frequency, and duration of ciprofloxacin for a specific disease per individual patient. In addition, to this, WHO DUE thresholds for ciprofloxacin were employed as standard criteria. WHO's criteria to evaluate drug use includes six parameters: correct indication in 90%, correct dose in 95%, correct frequency in 100%, and correct duration in 95%. Besides, drug interaction and contra-indication should be avoided in 90% and 100% of the participants, respectively. Finally, the outcome of the therapy is expected to be reported in 90% of the medical records [22].

### Operational definition

**Appropriate:** appropriateness was defined based on the four parameters, i.e. indication, dose, frequency, and duration of therapy. Therefore, appropriate utilization is correct in all four parameters.

**Inappropriate:** wrong prescriptions in one of the four- parameters, i.e. indication, dose, frequency, and duration of therapy.

**Indeterminate:** when there is missed/unrecorded information in one of the four-parameters, i.e. indication, dose, frequency, and duration of therapy.

**Ethical considerations:** ethical approval was obtained from the Institutional Health Research Ethics Review Committee (IHRERC), College of Health and Medical Science, Haramaya University. Permission letters were also received from respective hospital administrators (HFSUH, JH, FHPH, and SECIIIH) to conduct the study. The confidentiality of prescribers' and patients' information was maintained, and data obtained from the hospitals´ data was solely used for the analysis study.

## Results

**Sociodemographic characteristics of patients:** in this study, 522 medical records were incorporated with a male to female ratio of 1.03: 1. Ciprofloxacin was indicated in 478 (91.6%) participants age greater than eighteen years. Nearly half of the patients (n = 267, 51.1%) were within the age range between 25 and 49 years. Moreover, there was no record describing ciprofloxacin use in pregnant and lactating mothers (Table 1). Most of the medical records did not report on body mass index, height, and weight of the patients. Among patients treated with ciprofloxacin, about two-thirds (n = 348, 66.7%) were treated from the medical out-patient departments (OPD), followed by emergency OPD (n = 129, 24.7%), and the medical ward (n = 32, 6.1%) (Table 2). Complete blood count (CBC) was the most frequent laboratory investigation in half of the patients, followed by urinalysis.

**Table 1:** socio-demographic characteristics of patients who were on Ciprofloxacin therapy in selected governmental hospitals, Eastern Ethiopia from January 1^st^ - December 31^st^, 2016

		FHPH	HFSUH	JH	SECIIIH	Overall (%)
**Age (y)**	1-9	0	1	0	0	1 (0.2)
	10-18	2	22	19	0	43 (8.2)
	19-24	11	68	26	8	113 (21.7)
	25-49	35	98	79	55	67 (51.1)
	≥50	19	20	59	0	98 (18.8)
		**67**	**209**	**183**	**63**	**522**
**Sex**	Female	38	96	105	18	257 (49.2)
	Male	29	113	78	45	265 (50.8)
**Pregnancy**	Yes	0	0	0	0	0
	No	38	89	105	0	232
**Lactating**	Yes	0	0	0	0	0
	No	38	89	105	0	232

Hiwot Fana Specialized University Hospital (HFSUH), Federal Harar Police Hospital (FHPH), Jugel Hospital (JH) and Southeast Command III Hospital (SECIIIH), Harar, eastern Ethiopia

**Table 2:** wards where patients treated with ciprofloxacin in selected governmental hospitals, Eastern Ethiopia, within January 1^st^ - December 31^st^, 2016

Ward	FHPH	HFSUH	JH	SECIIIH	Overall (%)
Medical OPD	34	156	97	61	348 (66.7)
Emergency OPD	31	37	60	1	129 (24.7)
Medical Ward	0	8	23	1	32 (6.1)
Surgery	0	6	0	0	6 (1.1)
Pediatric OPD	2	2	0	0	4 (0.8)
Gyn /ob	0	0	3	0	3 (0.6)
	67	209	183	63	522

Hiwot Fana Specialized University Hospital (HFSUH), Federal Harar Police Hospital (FHPH), Jugel Hospital (JH) and Southeast Command III Hospital (SECIIIH), Harar, eastern Ethiopia

**Ciprofloxacin indications:** urinary tract infection (UTI), acute febrile illness (AFI), and typhoid fever (TF) were the common indications to which ciprofloxacin was prescribed (Table 3). Non-steroidal anti-inflammatory drugs (NSAIDs) such as diclofenac, paracetamol, tramadol, and ibuprofen were among the most frequently co-prescribed drugs accounting for 241 (46.2%) of all cases. Antimicrobial agents such as doxycycline, metronidazole, ceftriaxone, and amoxicillin were co-prescribed in one-fourth of participants (n=136, 26.1%) (Table 4).

**Table 3:** top ciprofloxacin indications in selected governmental hospitals, Eastern Ethiopia within January 1^st^ - December 31^st^, 2016

Indications	FHPH	HFSUH	JH	SECIIIH	Overall (%)
**UTI**	17	81	91	35	224 (42.9)
**AFI**	9	15	42	2	68 (13.0)
**Typhoid fever**	2	32	9	11	54 (10.4)
**Multiple**	11	26	8	6	51 (9.8)
**Age**	11	24	5	0	40 (7.7)
**LRTI**	3	10	0	0	13 (2.5)
**URTI**	4	1	3	0	8 (1.5)
**Others**	10	20	25	9	64 (12.3)
**Total**	**67**	**209**	**183**	**63**	**522**

Hiwot Fana Specialized University Hospital (HFSUH), Federal Harar Police Hospital (FHPH), Jugel Hospital (JH) and Southeast Command III Hospital (SECIIIH), Harar, eastern Ethiopia.

**Table 4:** top ciprofloxacin co-administered drugs in selected public hospitals (FHPH, HFSUH, JH, SECIIIH), Eastern Ethiopia within January 1^st^ - December 31^st^, 2016

Drugs	FHPH	HFSUH	JH	SECIIIH	Overall
**Diclofenac**	19	23	46	8	96 (13.8)
**Doxycycline**	15	34	23	0	72 (10.4)
**Paracetamol**	11	20	11	27	69 (9.9)
**Omeprazole**	9	19	25	11	64 (9.2)
**Tramadol**	8	18	17	10	53 (7.6)
**Ibuprofen**	5	2	5	11	23 (3.3)
**Ceftriaxone**	1	6	8	9	24 (3.5)
**Metronidazole**	4	8	9	0	21 (3.0)
**Amoxicillin**	14	3	2	0	19 (2.7)
**Cimetidine**	0	4	14	0	18 (2.6)
**Coartem**	0	5	3	8	16 (2.3)
**Glibenclamide**	0	3	3	9	15 (2.2)
**Tinidazole**	6	9	0	0	15 (2.2)
**Nefidepine**	2	3	0	6	11 (1.6)
**Insulin**	0	3	8	0	11 (1.6)
**Azithromycin**	5	5	0	0	10 (1.4)
**Dexamethasone**	5	0	3	0	8 (1.2)
**Others**	15	58	68	9	150 (21.6)
	119	223	245	108	**695**

Hiwot Fana Specialized University Hospital (HFSUH), Federal Harar Police Hospital (FHPH), Jugel Hospital (JH) and Southeast Command III Hospital (SECIIIH), Harar, eastern Ethiopia

**Evaluation of ciprofloxacin utilization:** ciprofloxacin utilization was primarily evaluated based on Ethiopian STG, considering the dose, indication, frequency, and therapy duration. Accordingly, drug therapy was found appropriate in less than one-third (n=159, 30%) of patients (Table 5). Incorrect duration (n =228, 95%) was identified as the primary reason for inappropriate utilization patterns. Moreover, the actual practice of ciprofloxacin utilization was evaluated against WHO drug use evaluation criteria with expected threshold points for the indication (90%), dose (95%), frequency (100%), and duration (95%). Besides, drug interaction and contraindication were to be avoided by 90% and 100% of the participants. Hence, 399 medication cards with the required information were considered for this particular evaluation. Indication, dose, and frequency were within the WHO criteria, but not the others (Table 6).

**Table 5:** appropriateness of ciprofloxacin indication in selected governmental hospitals, Eastern Ethiopia within January 1^st^ - December 31^st^, 2016

Hospitals	Appropriateness			
	Appropriate	Inappropriate	Indeterminate	Overall
**FHPH**	7	27	33	**67**
**HFSUH**	73	112	24	**209**
**JH**	61	74	48	**183**
**SECIIIH**	18	27	18	**63**
	**159 (30%)**	**240 (46%)**	**123 (24%)**	**522**

Hiwot Fana Specialized University Hospital (HFSUH), Federal Harar Police Hospital (FHPH), Jugel Hospital (JH) and Southeast Command III Hospital (SECIIIH), Harar, eastern Ethiopia

**Table 6:** comparison of actual utilization practice versus WHO set criteria for ciprofloxacin in selected governmental hospitals, Eastern Ethiopia within January 1^st^ - December 31^st^, 2016 (n = 399)

Criteria	Criteria expectation no (%)	Actual performance no (%)
**Indication**	359 (90)	385 (96.5)
**Dose**	379 (95)	399 (100.0)
**Frequency**	399 (100)	399 (100.0)
**Duration**	379 (95)	168 (42.1)
**Drug interaction**	359 (90)	234 (58.6)
**Contraindication**	399 (100)	363 (91.0)
**Outcome**	359 (90)	145 (36.3)

Hiwot Fana Specialized University Hospital (HFSUH), Federal Harar Police Hospital (FHPH), Jugel Hospital (JH) and Southeast Command III Hospital (SECIIIH), Harar, eastern Ethiopia

## Discussion

Ciprofloxacin is one of the commonly prescribed drugs in different parts of the world, owing to its broad-spectrum activity against a wide range of bacterial pathogens [5-7,23]. Besides, the drug has a favorable pharmacokinetic safety and efficacy profile [24]. On the contrary, the evaluation of its utilization pattern in resource-limited settings is minimal. Therefore, it is mandatory to evaluate the actual utilization pattern in such environments to pave the way in designing antimicrobial stewardship programs and antimicrobial use guidelines. By doing so, the emergence and spread of antimicrobial resistance and overall health costs can be managed. In this study, the higher patient flow was observed in outpatient settings dictating oral ciprofloxacin as a substitute for parenteral therapy in the treatment of bacterial infections [25]; likely reducing the costs associated with injectables and the hospital stay. CBC was the most frequently performed laboratory test in half of the participants, followed by urinalysis, indicating the routine practice of empirical therapy without any microbiological and susceptibility data. It is well established that ciprofloxacin is useful in treating the respiratory tract, urinary tract, reproductive system, and gastrointestinal tract infections, as well as many other bacterial infections caused by susceptible strains [2,26]. This study´s findings also showed UTI as the primary indication for ciprofloxacin utilization, followed by AFI, TF, and AGE. Similar utilization was reported from Boru Meda Hospital, South Wollo Zone of Ethiopia [27], and Dessie Referral Hospital (DRH), North East Ethiopia [4]. Though some literature also reported the UTI as one of the empirically treated infections by this drug, such practices might predispose for misuse of the antibacterial agent [28]. Additionally, one study also reported self-medication practices using this drug to treat UTI symptoms among communities as a shared practice [5]. Additionally, non-specific AFI was the second most common indication; implying treatment based on clinical presentation was the everyday management practice as there were no microbiological test results.

NSAIDs were among the highly co-prescribed drugs accounting for nearly half of all agents (52.7%), which justifies mutual symptomatic management related to infectious diseases. This is in concordant with the study conducted by Biru *et al*. who reported that NSAIDs were the most commonly co-indicated medications [27]. In addition, doxycycline, metronidazole, and ceftriaxone were the top antimicrobials co-prescribed. Evaluation for appropriateness of the utilization patterns based on Ethiopian STG revealed that ciprofloxacin therapy was appropriate in 30% of the patients. An incorrect length of use was identified as the primary contributor to inappropriate utilization. It is strongly believed that standards provide people and organizations with a basis for mutual understanding and are used as tools to facilitate communication and measurement. Accordingly, different nations at the country level and WHO as a global concern formulate criteria to evaluate practices of therapy using antimicrobials as one approach to promote therapeutic effectiveness and economically efficient prescribing [29]. However, this study revealed a gap in implementing the standards and inappropriate use of the drug, which is a crucial driver in antibiotic resistance.

It is widely accepted that antimicrobial resistance can potentially arise from early discontinuation of antibiotics following a temporary resolution of symptoms [30-32]. Though the efficacy of fluoroquinolones is dependent on concentration, optimum dosing schedules are recommended as it may best be defined according to their ability to minimize resistance in specific pathogenesis. Resistance to this drug arises in a stepwise fashion from low level to high level, depending on the acquisition of sequential mutations to the prime drug targets i.e. DNA gyrases [33] as antibiotics can trigger bacterial stress at sub-lethal concentrations [34]. To survive in these environmental conditions, cells have a repertoire of genes which either express or silence according to the need. Among the vast collection of genetically controlled networks, the SOS response is an inducible DNA repair system that allows bacteria to survive sudden increases in DNA damage [35]. Interestingly, SOS is also induced by sub minimal inhibitory concentrations of several antibiotics, including ciprofloxacin, which plays a pivotal role in the appearance and dissemination of resistance [36-38]. Both short duration and extended use of ciprofloxin is associated with the risk of adverse effects [39]. Suppression of intestinal flora by broad-spectrum antimicrobial agents, facilitate the risk of colonization or infection with resistant pathogen [33,40,41]. One limitation was incomplete data due to poor documentation and not recoding full drug-related information. Hence, the authors decided to report this problem as an indeterminate value, which accounted for 123 (24%) cases. Current advancements in medical science are based on information on the use of drugs and the management of diseases. Hence, poor medical record documentation practices by clinicians along with disorganized record keeping within the healthcare system can create an information gap among health professionals and patients potentially leading to misuse of drugs [42].

One of the fundamental professional responsibilities of health care providers are providing and documentation of drug information (DI). The primary purpose of delivering carefully evaluated, evidence-based recommendations to support specific medication-use practices is to enhance patient care quality, improve patient outcomes, and ensure the prudent use of resources [43]. Hence, documentation is crucial for appropriate patient care, highlights the value of services, demonstrates accountability, and provides a basis for quality assessment and performance improvement [44]. Furthermore, the actual practice of ciprofloxacin utilization was evaluated against WHO drug use evaluation criteria with expected threshold points for the indication (90%), dose (95%), frequency (100%), and duration (95%). Drug interaction and contraindication were to be avoided in 90% and 100% of the participants, respectively. The outcome of the therapy (negative culture, symptomatic improvement, or no treatment failure) was expected to be stated in 90% of the population. Hence, 399 medication cards with complete information were considered for this thorough evaluation and found that indication, dose, and frequency were in-line with the WHO threshold; however, the others were not. Antimicrobial resistance is one of the global agendas placing any achievements of the millennium development goals at risk and endangering the sustainable development goals. As a result, WHO is providing technical assistance to countries to aid in developing their national action plans and strengthening their health and surveillance systems; this assists the countries in preventing and managing antimicrobial resistance. Moreover, WHO is collaborating with partners to enhance evidence-based medicine and develop innovative responses to this global threat. Furthermore, it supports a standardized approach to collecting, analyzing, and sharing data related to antimicrobial resistance at an international level to inform decision-making, thus driving local, national and regional actions [45-47]. Hence, the result of this study could explain the level of the problem from international perspectives.

A similar study on ciprofloxacin use evaluation revealed none of the criteria were fulfilled [4], while another study showed that all the requirements were met as per the protocol [27]. Drug-drug interactions (DDIs) are a significant concern among patients receiving multidrug therapy. Careful attention and increasing knowledge frequently concerning co-indicated agents may render the framework for DDI prevention [48]. Different studies reported DDI to cause emergency room visits, hospital admissions, and contributed to mortality [49]. From this study, 41.4% of ciprofloxacin use had one or more potentially interacting drugs with analgesics (diclofenac, tramadol and ibuprofen) being the primary co-administered medication; followed by oral hypoglycemic agents, corticosteroids, and antacids. Co-administration of ciprofloxacin with analgesics may cause convulsions. Antacid containing Al or Mg may interfere with the absorption of ciprofloxacin. Drug interactions recorded in this study are higher than the threshold set for drug interactions by WHO (10%). In this study, the analysis for DDI considered those drugs with significant and moderate interactions [50]. It should be noted that there could have been possible interventions undertaken by healthcare providers to decrease DDI. So, the figure in this study shows only the potential DDI, not the actual outcome, as the review was retrospective in nature. Accordingly, this finding paves the way to increase knowledge concerning probable DDI and methods to prevent and mitigate the effects. Other studies reported potentially interacting with ciprofloxacin as a common threat similar to our research findings [4,27].

When contraindicated, prescribing drugs may be justified at times if the benefits versus risks are considered. Ciprofloxacin was used despite contraindications in 8.4% (n=44) cases, which included children less than eighteen years and diabetic patients. This result is higher than the WHO threshold, which mandates 0% use against contraindications. Such practice was also reported 5% in North East [51] and 10% in South Wollo Ethiopia [27]. Inappropriate length of therapy was the major problem identified by this study. Only 42.1% of ciprofloxacin uses were within the duration of therapy guidelines which was far below the threshold set (95%) by WHO. Similar findings were reported from other studies conducted in the country [27]. A systematic review and meta-analysis conducted on antimicrobial resistance in West Africa reported the level of resistance to ciprofloxacin was increasing at an alarming rate [52], suggesting that appropriate utilization as per duration of treatment stated for a particular disease recommended in the STGs and formularies is a mandatory step to alleviate the problem. Antimicrobial resistance in bacterial pathogenesis is a worldwide challenge associated with high morbidity and mortality [23,52,53]. This effect is prevalent in resource-limited countries such as Ethiopia, resulting in limited options in treating infectious diseases as resistant isolates are now frequently encountered in the country [13].

**Strength and limitation:** ciprofloxacin use evaluation was conducted considering the patient characteristics of four governmental hospitals enabling us to compare results from various perspectives and reflective of the utilization practice patterns of ciprofloxacin in the study area. In addition, the retrospective review of patient medical records posed less risk of bias, as the data were abstracted directly from records. However, the retrospective nature of the study might not reflect the drugs current usage. Additionally, due to using secondary data sources, another limitation encountered was poor documentation.

## Conclusion

Almost equal involvement of both genders was noted, and the majority were treated at medical and emergency OPD. The most frequent laboratory investigation undertaken was the CBC in half of the participants. The major indications for ciprofloxacin usage were UTI, AFI, TF, and AGE. NSAIDs and antimicrobial agents were among the most frequently co-administered drugs with ciprofloxacin. Based on Ethiopian STG, drug therapy was declared appropriate in 30% of treated patients. The incorrect length of treatment was identified as a major contributor to inappropriateness. Moreover, ciprofloxacin utilization was compared with the WHO thresholds; indication, dose, and frequency were within the threshold limits. Future studies need to address the susceptibility patterns of the responsible isolates of the pathogenic organisms for the top ciprofloxacin indications to decide on treatment strategies. From this, effective antimicrobial stewardship programs can be designed for healthcare facilities.

### What is known about this topic

- One of the top drugs being used in the country;
- Drug resistance for ciprofloxacin is gradually increasing.

### What this study adds

- Inappropriate utilization pattern was found as per Ethiopian standard treatment guideline and World Health Organization set criteria and this might predispose for drug resistance;
- The main reason for inappropriate utilization was the incorrect length of treatment;
- Poor practices of recording essential drug and patient-related information on the medication card by clinicians along with disorganized documentation.
